# Field-Deployable Determinations of Peroxide Index and Total Phenolic Content in Olive Oil Using a Promising Portable Sensor System

**DOI:** 10.3390/s23115002

**Published:** 2023-05-23

**Authors:** Marco Grossi, Alessandra Bendini, Enrico Valli, Tullia Gallina Toschi

**Affiliations:** 1 Department of Electrical Energy and Information Engineering “Guglielmo Marconi” (DEI), Alma Mater Studiorum—Università di Bologna, 40126 Bologna, Italy; 2 Department of Physics and Astronomy “Augusto Righi” (DIFA), Alma Mater Studiorum—Università di Bologna, 40126 Bologna, Italy; 3 Department of Agricultural and Food Sciences (DISTAL), Alma Mater Studiorum—Università di Bologna, 47521 Cesena, Italy; alessandra.bendini@unibo.it (A.B.); enrico.valli4@unibo.it (E.V.); 4 Interdepartmental Centre for Industrial Agrofood Research, Alma Mater Studiorum—Università di Bologna, 47521 Cesena, Italy; tullia.gallinatoschi@unibo.it; 5 Department of Agricultural and Food Sciences (DISTAL), Alma Mater Studiorum—Università di Bologna, 40126 Bologna, Italy

**Keywords:** portable sensors, optical sensors, olive oil analysis, total phenolic content, peroxide index

## Abstract

Useful information about the oxidative stability of a virgin olive oil in terms of oxidation products and antioxidant compounds can be obtained by analyzing the peroxide index (PI) and total phenolic content (TPC), respectively. These quality parameters are usually determined in a chemical laboratory using expensive equipment, toxic solvents, and well-trained personnel. This paper presents a novel portable sensor system for in the field and rapid determination of PI and TPC that is particularly suited in the case of small production environments that cannot afford an internal laboratory for quality control analysis. The system is small, can be powered by both USB ports and batteries, is easy to operate, and integrates a Bluetooth module for wireless data transmission. It estimates the PI and TPC in olive oil from the measurement of the optical attenuation of an emulsion between a reagent and the sample under test. The system has been tested on a set of 12 olive oil samples (eight for calibration and four for validation), and the results have shown how the considered parameters can be estimated with good accuracy. The maximum deviation from the results obtained with the reference analytical techniques is 4.7 meq O_2_/kg in the case of PI and 45.3 ppm in the case of TPC for the calibration set, while it is 14.8 meq O_2_/kg in the case of PI and 55 ppm in the case of TPC for the validation set.

## 1. Introduction

Olive oil is highly appreciated for its sensory attributes and beneficial effects on human health [1]. The quality of olive oil is defined by a set of parameters measured by chemical analyses as well as sensory analyses carried out by a panel of experts (the Panel test).

An important parameter that defines the quality of olive oil in terms of primary oxidative products is the peroxide index (PI), expressed as milliequivalents of active oxygen per kilogram of oil (meq O_2_/kg oil). If, during storage, the oil is exposed to light or the storage temperature is not adequate, oxidation is promoted, and the PI starts to increase [2]. On the other hand, very useful information can be taken from the total phenolic content (TPC) related to powerful antioxidants naturally present in virgin olive oils. In fact, phenolic compounds that act as free radical traps, contributing to protecting our body from some health diseases and providing anti-cancer activity [3], are also responsible for the product’s shelf life [4], as for the bitterness and pungency that are known as positive sensory characteristics of the product. The reference analytical techniques to measure PI and TPC in olive oil are manual titration [5] and liquid-liquid extraction followed by a spectrophotometric assay [6], respectively. These are chemical analyses that must be carried out in a laboratory by trained personnel using toxic solvents and standards. Moreover, the determination of TPC requires specific instrumentation and time-consuming procedures.

In the case of small olive oil mills and packaging centers that cannot afford an internal laboratory for quality control analyses, the sample to be tested must be shipped to an external laboratory, with high costs and a long response time. Thus, there is a need for rapid and low-cost solutions for the analysis of olive oil quality parameters that can be carried out in the field in a real production environment. In recent years, many field-deployable analytical approaches have been proposed for the quality and purity assessments of oils and fats. Some examples are: the determination of solid fat content in fats and oils by optical attenuation analysis [7,8]; the determination of iodine value of vegetable oils using a smartphone camera [9]; the measurement of total polyphenol concentration using a smartphone camera [10]; the quality analysis and determination of free acidity of olive oil using a portable sensor system [11,12]; the rapid detection of olive oil blends using a paper-based portable microfluidic platform [13]; the identification of edible oil storage period using a portable electronic nose [14]; the detection of food fraud in extra virgin olive oil using a portable hyphenated photonics sensor [15].

In this paper, a portable electronic sensor system for the determination of PI and TPC in olive oil is presented. The instrument is small, light-weighted, and battery-operated to allow in-field olive oil analysis. The instrument’s working principle is based on optical attenuation measurements of an emulsion of the oil sample with a chemical reagent. The portable sensor system has been tested outside a laboratory, and the results have shown a good correlation with the oil quality parameters determined by the reference analytical techniques.

## 2. Materials and Methods

### 2.1. Olive Oil Samples

The measurements have been carried out on a set of 12 olive oil samples (8 samples for calibration and 4 samples for validation), featuring a PI value that ranges from 5.3 to 31.9 meq O_2_/kg oil and a TPC value that ranges from 69.5 to 496.1 ppm.

For each investigated sample, PI and TPC have been determined by laboratory analysis using the reference methods. In the case of PI, the value has been determined according to the IOC and EU standard reference methods with starch as an indicator and sodium thiosulfate (Na_2_S_2_O_3_) as a titrant. In the case of TPC, the value has been determined according to the spectrophotometric assay based on the Folin–Ciocalteu reagent as proposed by Singleton and Rossi [16]. In particular, the phenolic fraction was extracted from the olive oil samples, and then, after the reaction with the specific reagent in alkaline medium, the phenolic content was detected at 750 nm and quantified, as reported in Reboredo-Rodrìguez et al. [17]. The values of PI and TPC determined with the reference methods have been used to evaluate the accuracy of the proposed embedded sensor system.

### 2.2. Solvents and Reagents

The reagents and chemicals used in this study, including methanol, sodium thiosulfate, Folin–Ciocalteu reagent, sodium carbonate, and ferrous oxidation-xylenol orange (FOX), were of analytical grade and were purchased from Sigma-Aldrich (St. Louis, MO, USA) or Merck (Readington, NJ, USA).

### 2.3. Measurement Principle

The olive oil parameters considered in this work, specifically peroxide index and total phenolic content, were estimated with the proposed portable sensor system using optical attenuation as the sensing principle. The olive oil under test was mixed with a suitable reagent to create an emulsion, and its optical attenuation was evaluated for a radiation source of known wavelength.

In the case of the peroxide index determination, the selected chemical chromophore is obtained by mixing 8 mL of FOX reagent with 7 mL of distilled water, and peroxides are detected according to the reaction:

(1) $$Fe^{2+}+ROOH\to Fe^{3+}+RO+OH^{-}$$

where *Fe^3+^* ions oxidize the dye xylenol orange, which produces a chromophore with strong absorption in the wavelength range around 580 nm.

In the case of the total phenolic content determination, the selected chemical reagent is obtained by mixing 13 mL of distilled water with 1 mL of Folin–Ciocalteu reagent and 1 mL of sodium carbonate Na_2_CO_3_ 15%. In the presence of reducing compounds, such as the phenolic compounds, the acids present in the reagent are reduced to tungsten and molybdenum oxides, resulting in an optical attenuation in the wavelength range 500–900 nm with higher sensitivity in the near infrared wavelength range (680–900 nm).

Preliminary measurements were carried out using laboratory instrumentation to work out the optimal conditions in terms of radiation wavelength and reagent/oil sample ratio. An experimental setup was built, composed of a Falcon vial containing the emulsion, a LED, and a photodiode. The LED was supplied with a square-wave voltage using a benchtop function generator (Agilent 33210A, Agilent Technologies, Santa Clara, CA, USA), and the current through the photodiode was converted to voltage and acquired using a National Instruments data acquisition device (USB-6003, National Instruments, Austin, TX, USA). LabVIEW programs were developed to acquire, process, and save the measured data on the PC hard disk for further analysis. In the case of peroxide index determination, the reaction of FOX reagent with peroxide compounds produces a strong absorption peak between 550 and 600 nm [18]. LEDs with different wavelength peaks (560, 570, 580, 590, and 600 nm) were tested, and the best results were obtained in the case of peak wavelength 590 nm. Different reagent/sample ratios were also tested (0.5, 1, 2, and 5 mL of sample in 15 mL of reagent), and the best result was obtained in the case of 0.5 mL of olive oil. Similarly, in the case of total phenolic content determination, the reaction of the Folin–Ciocalteu reagent with phenolic compounds produces a strong absorption in the wavelength range 680–900 nm [19]. LEDs with different wavelength peaks (700, 750, 800, 850, and 900 nm) and different reagent/sample ratios (0.5, 1, 2, and 5 mL of sample in 15 mL of reagent) were tested, and the best results were obtained in the case of an LED with a peak wavelength of 850 nm and 0.5 mL of olive oil.

In the following, the emulsion was created by mixing 15 mL of the reagent with 0.5 mL of the olive oil under test.

### 2.4. Portable Sensor System

**Figure 1:**
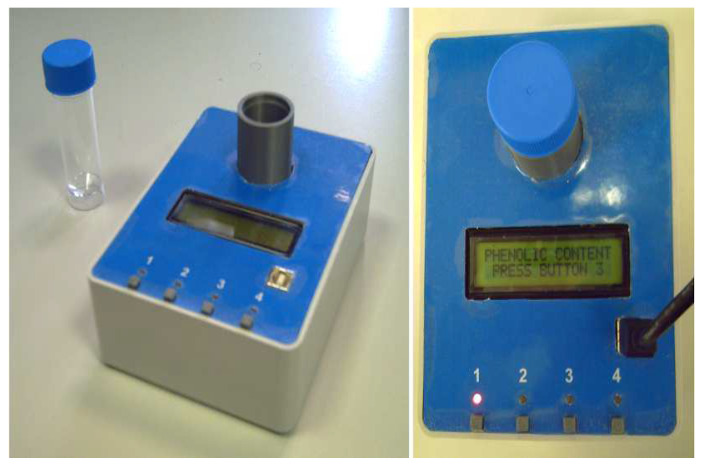
Picture of the proposed portable sensor system for the determination of peroxide index and total phenolic content in olive oil.

The portable sensor system for in the field determination of peroxide index and total phenolic content is presented in Figure 1. It is composed of a plastic case (11 cm × 15 cm × 5 cm) integrated with four buttons for user interaction and a 16 × 2 LCD display, a Polylactic Acid (PLA) structure (built with a 3D printer, MakerBot Replicator Z18, MakerBot Industries, New York City, NY, USA) devoted to hosting the vial with the emulsion as well as the LEDs and photodiodes, and an electronic board, designed by the research team using KiCad 5.1.2 [20], to control the LEDs and measure the currents in the photodiodes.

**Figure 2:**
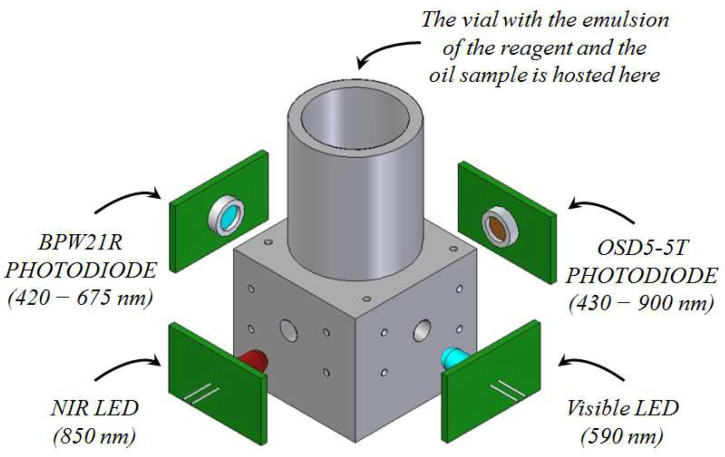
Picture of the PLA structure devoted to the host of the emulsion and the LEDs/photodiodes used for the optical attenuation measurements.

The PLA structure built with the 3D printer is presented in Figure 2. The vial containing the emulsion to be analyzed is hosted in a cylindrical cavity, and two couples of LEDs/photodiodes are integrated in the structure for the optical attenuation measurements. The LED generates the radiation that is transmitted through the emulsion, and the attenuated radiation emerging from the emulsion is detected by the photodiode. In the case of peroxide index measurements, the visible LED with a peak wavelength of 590 nm and the photodiode (BPW21R, Vishay Intertechnology, Malvern, PA, USA) with a wavelength sensitivity range of 420–675 nm is used. In the case of total phenolic content measurements, the near-infrared (NIR) LED with a peak wavelength of 850 nm and the photodiode (OSD5-5T, Centronic, Croydon, UK) with a wavelength sensitivity range of 430–900 nm is used.

The designed electronic board is based on the 32 bit low-power microcontroller STM32L152VCT6 by ST Microelectronics and integrates a Bluetooth class-2 module (SPBT2632C2A by ST-Microelectronics, Geneva, Switzerland) that allows wireless data transmission in addition to data presentation on the LCD display and wired data transmission using the USB port.

**Figure 3:**
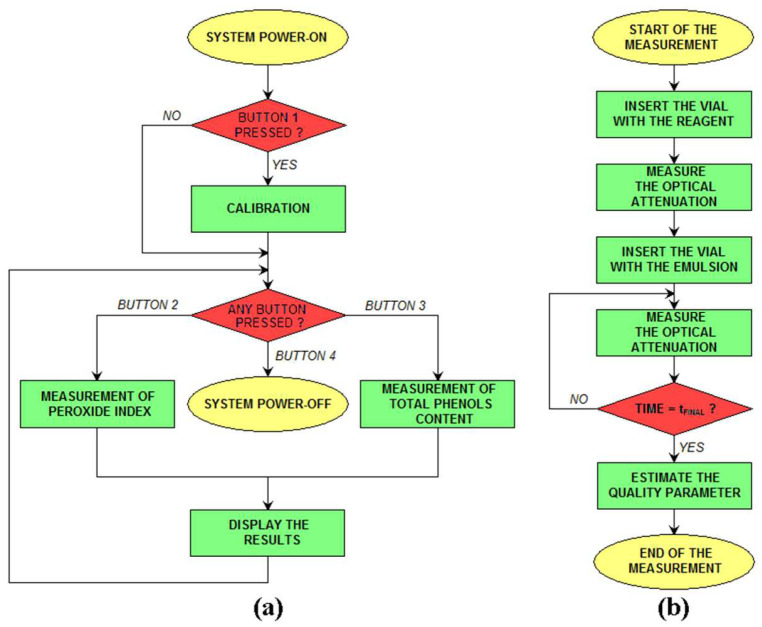
Flowchart of the general procedure for olive oil analysis (**a**) and single measurement steps (**b**) using the proposed sensor system.

The procedure for olive oil analysis using the proposed sensor system is presented in the flowcharts in Figure 3a,b. When the system is powered on, if button 1 is pressed, the system enters a calibration procedure where the LED intensity is changed to produce a target output value in the case of an empty vial. The value of the LED intensity is then stored on an EEPROM memory integrated in the microcontroller. Then, the system waits for the user to select the action (button 2: measurement of PI; button 3: measurement of TPC; and button 4: power off the system). After the selection, the measurement is carried out, and the results are displayed on the LCD screen. The procedure for a single measurement is presented in the flowchart in Figure 3b. Initially, the LCD screen informs the user to insert a vial with only the reagent, and a measurement of the optical attenuation is carried out to check that the optical properties of the reagent are correct (i.e., the reagent is not degraded). Then, the system asks the user to insert the vial with the emulsion created with the reagent and the oil (the procedure is discussed in Section 2.3), and measurements of optical attenuation are carried out at time intervals of 5 s for a total time of 900 s (15 min). Then, the oil parameter of interest is estimated on the measured value at a particular time (600 s for PI and 500 s for TPC, as will be discussed in Section 3), and such a value is displayed on the LCD screen. Even if the system estimates the quality parameter of interest based on the measurement at a fixed time, the entire dataset (optical attenuation vs. time) is recorded and can be downloaded to a PC for further data processing.

**Figure 4:**
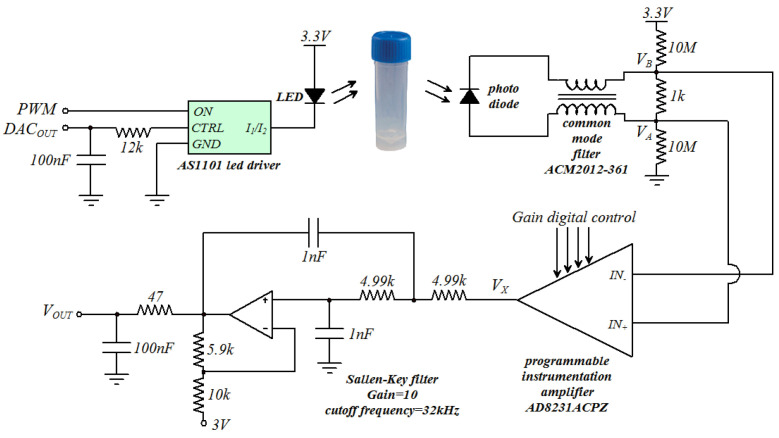
Schematic of the circuit for the optical attenuation measurements.

**Figure 5:**
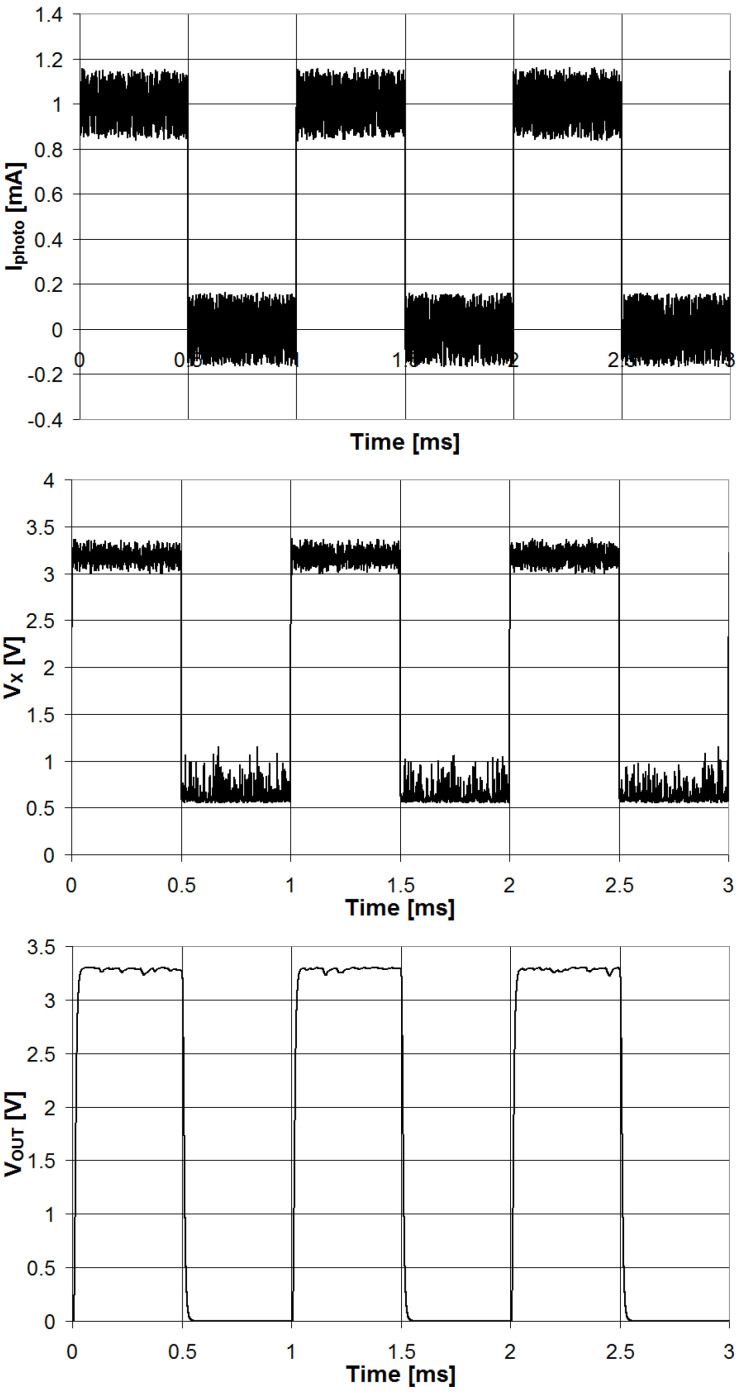
Waveforms of the signals of the circuit for the optical attenuation measurements obtained with LTSpice simulations.

The circuit for the measurement of the emulsion’s optical attenuation is shown in Figure 4. The chip AS1101 (ams-OSRAM AG, Premstaetten, Austria, EU) is an LED driver used to control the two LEDs used in the system. The analog input CTRL is generated using the 12 bit DAC integrated in the microcontroller and used to control the intensity of the current flowing in the LED when in the ON state. The input ON is fed with a PWM signal (frequency 1 kHz, duty-cycle 50%) generated by the microcontroller to drive the LED ON and OFF according to the PWM signal. The LED generates a pulse of light with a frequency of 1 kHz that is transmitted through the vial containing the emulsion. The light emerging from the vial is detected by the photodiode, which generates a pulsed current that is then converted to voltage, amplified with the programmable instrumentation amplifier AD8231ACPZ (Analog Devices, Wilmington, MA, USA) and filtered using a Sallen–Key low-pass filter with a cutoff frequency of 32 kHz. The circuit has been simulated using LTSpice XVII by Analog Devices [21], and the signal waveforms are presented in Figure 5.

### 2.5. Statistical Analysis

The measured data have been processed using Microsoft Excel 2010 and its statistical add-on package, XLSTAT 2016. The olive oil quality parameters of interest (PI and TPC) have been estimated by fitting the light attenuation values measured by the sensor system with the quality parameters determined using reference laboratory techniques.

The accuracy of the estimated values of quality parameters has been evaluated using the mean square error (*MSE*), which gives information on the mean quadratic discrepancy between the values obtained with the reference techniques and the values predicted with the proposed sensor system. *MSE* can be calculated with the following equation:

(2) $$MSE=\frac{1}{N}\times \sum_{i=1}^{N}{\left(Y_{i}-X_{i}\right)}^{2}$$

where *N* is the number of tested samples, *Y_i_* is the estimated quality parameter for sample *i*, and *X_i_* is the quality parameter obtained with the reference techniques for sample *i*.

## 3. Results and Discussion

### 3.1. Determination of the Optimal Measurement Time

**Figure 6:**
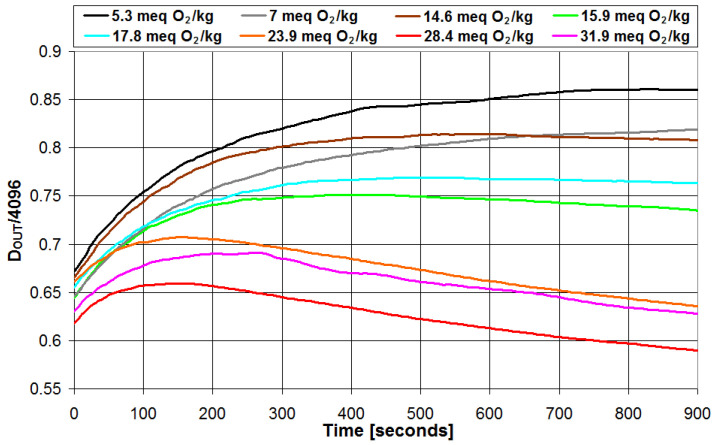
Values of the intensity of the radiation transmitted through the emulsion plotted vs. time in the case of peroxide index measurements.

**Figure 7:**
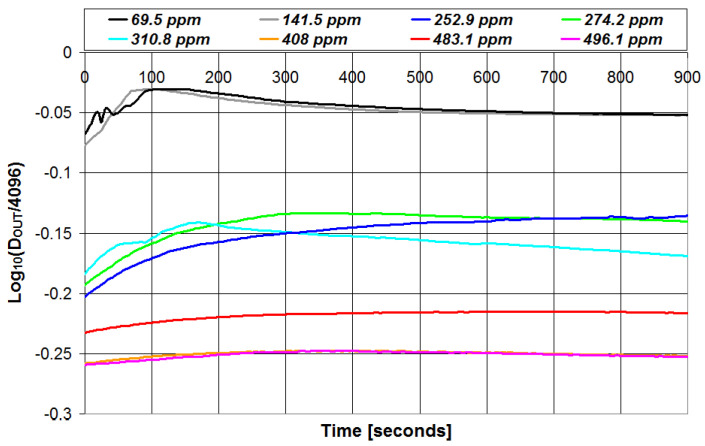
Values of the intensity of the radiation transmitted through the emulsion plotted vs. time in the case of total phenolic content measurements.

A set of eight olive oil samples has been used to calibrate the proposed portable instrument after the analytical parameters (PI and TPC) of each sample have been determined using the reference laboratory techniques. The intensity of the radiation transmitted through the emulsion has been monitored at time intervals of 5 s for a total of 900 s. The results are presented in Figure 6 and Figure 7 for the PI and TPC, respectively, in the case of all 8 samples. In the figures, the intensity of the transmitted radiation is reported with the parameter *D_OUT_*, which represents the digital value (between 0 and 4095) of the peak-to-peak voltage of the signal V_OUT_ in Figure 4 when measured with the 12 bit ADC integrated in the microcontroller.

As can be seen, for both PI and TPC measurements, the intensity of the transmitted light presents a rapid change in the first minutes after the emulsion creation and then tends to saturate after 9–10 min. These are the results of the reaction between the chemical reagent and the olive oil under test, which took some time to complete.

To evaluate the best trade-off between measurement speed and accuracy in the estimation of quality parameters, different measurement times (0 s, 100 s, 200 s, 300 s, 400 s, 500 s, and 600 s) have been considered and the corresponding parameters estimated in each case. Measurement times higher than 600 s are not considered since we have defined 10 min as the maximum reasonable measurement time for quick in the field olive oil analysis. Moreover, we have checked that the potential improvement in accuracy due to longer measurements is negligible and lower than the uncertainty associated with repeated measurements. Then, the accuracy of the estimation is evaluated using the *MSE* parameter. The *MSE* parameter is calculated as follows: for each measurement time, the calibration equation that correlates the optical attenuation data with the quality parameter of interest determined with the reference technique is calculated, and such a calibration equation is used to estimate the values of the quality parameter of interest. Then, the *MSE* value is calculated from the analytical parameter determined with the reference technique and its estimated value using Equation (2). The results are presented in Table 1. As can be seen, *MSE* strongly decreases with measurement time in the first 5 min (300 s) and then slightly decreases and saturates. The best accuracy (i.e., lowest value of *MSE*) is obtained after 600 s for PI and after 500 s for TPC. Thus, in the following, the olive oil parameters have been determined from the value of *D_OUT_* measured at 600 s for PI and at 500 s for TPC.

### 3.2. Accuracy of the Estimated Quality Parameters

**Figure 8:**
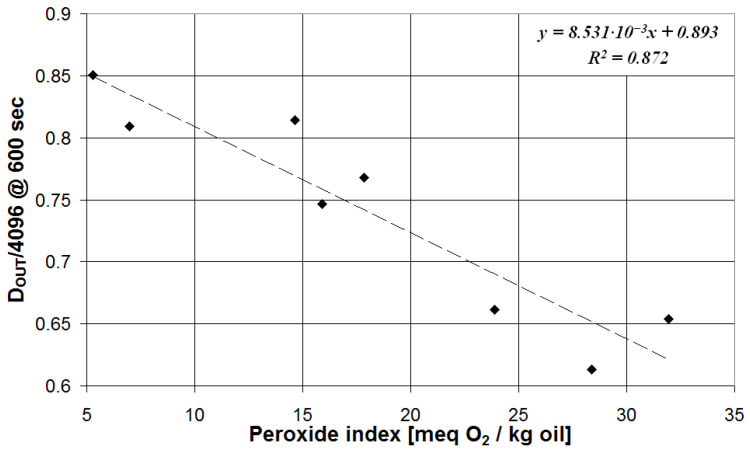
System response after 600 s plotted vs. the olive oil PI determined with the reference method.

**Figure 9:**
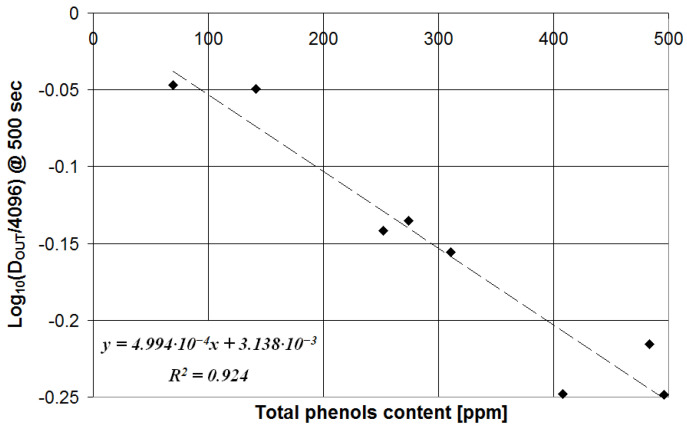
System response after 500 s plotted vs. the olive oil TPC determined with the reference method.

The value of *D_OUT_* for the target measurement time (600 s for PI and 500 s for TPC) has been plotted vs. the corresponding parameter determined with the reference technique for each olive oil sample from the calibration set. The results are presented in Figure 8 for the measurement of PI and in Figure 9 for the measurement of TPC.

As can be seen in Figure 8, the value of *D_OUT_* at 600 s presents a linear correlation with the PI determined using the reference technique, and the corresponding calibration equation is defined by the following:

(3) $$\mathrm{PI}=10^{3}\times \frac{\left(D_{OUT}/4096\right)-0.893}{8.531}$$

and the determination coefficient is R^2^ = 0.872.

In the case of TPC measurements, instead, the correlation between *D_OUT_* at 500 s and the TPC value determined with the reference technique is logarithmic, as can be seen in Figure 9, and the corresponding calibration equation (R^2^ = 0.924) is defined by the following:

(4) $$\mathrm{TPC}=10^{4}\times \frac{\log_{10}\left(D_{OUT}/4096\right)-3.138\times 10^{-3}}{4.994}$$

The calibration Equations (3) and (4) have been used to estimate the values of PI and TPC, and such values (mean value and error from triplicate measurements) have been compared with the results of the reference techniques in Table 2 and Table 3, respectively.

As can be seen, in both cases, the estimated value features good accuracy. In particular, in the case of PI measurements, the maximum deviation between the mean value calculated with the proposed system and the reference analytical protocol is 4.7 meq O_2_/kg oil, and all investigated samples are classified in the same quality category as defined by the reference method (the threshold value for edible virgin olive oil is 20 meq O_2_/kg). In fact, as reported in Table 2, for the proposed and reference methods, the same quality grade is defined, specifically the samples coded three, six, and eight are classified as lampante olive oil or not edible instead of the other five samples as edible olive oil (being the same limit fixed for both extra virgin and virgin quality grades).

In the case of TPC measurements, the maximum deviation between the value calculated with the proposed system and the reference technique is 45.3 ppm, and the proposed system is able to detect the usual concentration range of phenolic compounds in the oil [22]. Specifically, the samples coded as three and six are characterized by a low content of phenolic compounds (below 200 mg per kg of oil) for both methods, as are other samples with a medium content (ranging from 200 to 500 mg per kg of oil).

To investigate the effect of matrix variations among different olive oils on the accuracy of the estimated quality parameters, the equations obtained with the calibration group of eight olive oil samples have been used to estimate the analytical parameters on a different set of four olive oil samples. The results are presented in Table 4 for the PI and in Table 5 for the TPC.

As can be seen in Table 4, all validation samples are classified in the same quality category as defined by the reference method (edible virgin olive oil with a PI < 20 meq O_2_/kg for samples A, B, and C and lampante olive oil with a PI > 20 meq O_2_/kg for sample D). In the case of sample D, there is a significant overestimation of PI that could be explained by the presence of interfering compounds from secondary oxidation.

Similarly, for the case of TPC, shown in Table 5, the concentration range of phenolic compounds is correctly estimated for all samples (low content of phenolic compounds for samples A and D and medium content of phenolic compounds for samples B and C).

## 4. Conclusions

The paper presents a novel sensor system for the estimation of peroxide index (PI) and total phenolic content (TPC) in virgin olive oils. The sensor system is of small size, easy to operate, can be powered using a USB port or batteries, and integrates a Bluetooth module for wireless data transfer. It estimates the parameters of interest by measuring the optical attenuation of an emulsion between a chemical reagent and the olive oil sample.

The proposed system is particularly interesting in the case of olive oil mills and small packaging centers that cannot afford an internal laboratory for quality control analysis. It can perform measurements in the field and be used by operators without specific training.

The sensor system has been tested on a set of 12 olive oil samples (eight for calibration and four for validation) featuring different values of the quality parameters. The results have shown that accurate determination of PI and TPC is feasible. The maximum deviation from the results obtained with the reference techniques is 4.7 meq O_2_/kg in the case of PI and 45.3 ppm in the case of TPC for the calibration set, while it is 14.8 meq O_2_/kg in the case of PI and 55 ppm in the case of TPC for the validation set. To confirm the herein presented promising results, a set composed of a higher number of olive oils will be assessed.

## Figures and Tables

**Table 1:** *MSE* values for different measurement times in the case of PI and TPC determination of the olive oil sample set.

Measurement Type	0 s	100 s	200 s	300 s	400 s	500 s	600 s
Peroxide index	98.9	31.8	21.6	17.5	13.5	12.6	11.7
Total phenolic content	3403.7	2493.3	2046.2	1840.3	1725.8	1699.0	1723.7

**Table 2:** Olive oil peroxide index values (expressed in meq O_2_/kg) estimated with the proposed system and obtained with the reference technique in the case of the calibration set of eight olive oil samples.

Olive Oil Sample	PI (Proposed Sensor System)	PI (Reference Technique)
#1	3.0 ± 1.9	5.3 ± 0.4
#2	15.7 ± 1.5	15.9 ± 1.1
#3	30.2 ± 2.7	28.4 ± 2.0
#4	8.2 ± 1.7	7.0 ± 0.5
#5	13.1 ± 1.7	17.8 ± 1.2
#6	27.7 ± 0.5	23.9 ± 1.7
#7	13.9 ± 4.5	14.6 ± 1.0
#8	29.9 ± 1.7	31.9 ± 2.2

**Table 3:** Olive oil total phenolic content values (expressed in ppm) estimated with the proposed system and obtained with the reference technique in the case of the calibration set of eight olive oil samples.

Olive Oil Sample	TPC (Proposed Sensor System)	TPC (Reference Technique)
#1	443.5 ± 18.0	483.1 ± 19.3
#2	261.8 ± 2.4	274.2 ± 11.0
#3	105.0 ± 12.2	141.5 ± 5.7
#4	440.6 ± 50.0	408.1 ± 16.3
#5	294.1 ± 11.4	310.8 ± 12.4
#6	75.9 ± 11.8	69.5 ± 2.8
#7	497.2 ± 5.4	496.1 ± 19.8
#8	297.3 ± 20.0	251.9 ± 10.1

**Table 4:** Olive oil peroxide index values (expressed in meq O_2_/kg) estimated with the proposed system and obtained with the reference technique in the case of the validation set of four olive oil samples (A–D).

Olive Oil Sample	PI (Proposed Sensor System)	PI (Reference Technique)
#A	4.1 ± 1.5	7.6 ± 0.5
#B	10.3 ± 2.1	17.8 ± 1.2
#C	17.2 ± 1.8	15.9 ± 1.1
#D	45.8 ± 5.6	31.0 ± 2.2

**Table 5:** Olive oil total phenolic content values (expressed in ppm) estimated with the proposed system and obtained with the reference technique in the case of the validation set of four olive oil samples (A–D).

Olive Oil Sample	TPC (Proposed Sensor System)	TPC (Reference Technique)
#A	124.2 ± 12.3	140.5 ± 5.6
#B	421.7 ± 23.7	387.4 ± 15.5
#C	229.8 ± 7.4	229.9 ± 9.2
#D	120.4 ± 9.8	175.4 ± 7.0

## Data Availability

Not applicable.
